# Enabling a fast annotation process with the Table2Annotation tool

**DOI:** 10.5808/GI.2020.18.2.e19

**Published:** 2020-06-15

**Authors:** Pierre Larmande, Kazim Muhammed Jibril

**Affiliations:** 1 DIADE, Univ. Montpellier, IRD, Montpellier, France; 2 ICTLab, USTH, Hanoi, Vietnam

**Keywords:** bioinformatics, ontologies, semantic annotation

## Abstract

In semantic annotation, semantic concepts are linked to natural language. Semantic annotation helps in boosting the ability to search and access resources and can be used in information retrieval systems to augment the queries from the user. In the research described in this paper, we aimed to identify ontological concepts in scientific text contained in spreadsheets. We developed a tool that can handle various types of spreadsheets. Furthermore, we used the NCBO Annotator API provided by BioPortal to enhance the semantic annotation functionality to cover spreadsheet data. Table2Annotation has strengths in certain criteria such as speed, error handling, and complex concept matching.

**Availability:** GitHub: https://github.com/pierrelarmande/ontology-project.

## Introduction

Semantic annotation has been defined in various ways by various authors, but these definitions are all similar and reflect a single clear purpose. For instance, Oliveira and Rocha [1] defined semantic annotation as the process in which semantic concepts are linked to natural language. Liao et al. [2] defined semantic annotation as methods of describing resources (texts, images ...) with metadata where the meaning has been specified in an ontology. According to Oliveira and Rocha [1] semantic annotation can be seen as a methodology of adding metadata—comprising classes, properties, relations, and instances (i.e., the concepts of an ontology)—to web resources to be able to give or allocate semantics. Summarizing all of these definitions, we can simply state that semantic annotation is a way of matching resources to ontologies.

To make this point clearer, take this example of the text “.*days to flowering…*” With the help of semantic annotation, we would be able to match this text to the ontology concept “*days to flowering trait*” from the Trait Ontology [3], which has the concept ID of TO:0000344.

Semantic annotation helps to boost the ability to search and access resources. It is also a step towards data FAIRification [4]. According to Jovanovic and Bagheri [5], semantic annotation can be used in information retrieval to expand the queries from the user with some ontology terms and also to provide a grouping of documents retrieved based on specific content. Biomedical resources contain numerous abbreviations in the texts, sometimes with different meanings, which makes it hard to perform comprehensive searches. Semantic annotation helps to disambiguate these abbreviated terms based on the way they appear in a certain context.

In this paper, we sought to identify ontological concepts in scientific texts. This could be seen as an ontology matching process, in which natural language texts are matched with concepts. There are already some existing web services and tools that use semantic annotation for ontology matching, as have been evaluated by Oliveira and Rocha [1]. However, few of these tools handle spreadsheet data as text input. We developed the Table2Annotation tool with that purpose because there is a need for such a tool in the life sciences community, which produces extensive experimental data in spreadsheets. Semantic annotations will facilitate more complex analyses across several datasets.

The paper is organized as follows. Section 2 defines the challenges of semantic annotation. Section 3 presents an overview of Table2Annotation. Section 4 analyzes the results of semantic annotation through some examples. Section 5 concludes the manuscript.

## Semantic Annotation Challenges

Semantic annotation has some benefits, but some challenges are also faced during the annotation of biological texts or other resources. Some of these challenges, as also described in previous research [6-8], are follows:

‒ Word sense disambiguation: It is necessary to determine the correct meaning of a word as used in a sentence when a word has multiple meanings.

‒ Spelling/grammatical error identification: Correcting spelling or grammar in biomedical texts is very important. Spelling and grammar errors cause ambiguity in already sparse text.

‒ Discontinuous entities: Entities can be composed of multiple words in a discontinuous span. For example, “*drought and salinity tolerance*” means “*drought tolerance and salinity tolerance,*” but in this case we might only have matching for “salinity tolerance.”

‒ Gene/protein disambiguation: In the biomedical context, all proteins have associated genes, often with the same name, making it difficult to annotate texts dealing with genes and proteins.

‒ Detection of name variants: Variations of entity naming can take many forms, thereby complicating annotations. For example, abbreviations and shorthand texts are difficult to normalize with ontological concepts.

The challenges faced in annotation can be tackled by two approaches, which can be also combined. The first one is the term-to-concept matching method, which involve matching some parts of the provided text to structured knowledge databases, dictionaries, or vocabularies. However, it is difficult to maintain comprehensive lexicons to be used for annotation. The second approach is machine learning, which involves creating annotators for specific purposes and usage instead of more general ones [5].

Of particular note, the third challenge (discontinuous entities) can be tackled by creating algorithms that can transform texts with conjunctions like “*and*” or “*or.*” Thus, in the example of “*drought and salinity tolerance,*” an algorithm could transform this phrase to “*drought tolerance and salinity tolerance*” before the annotation process.

Although these are good solutions to tackle some challenges, some drawbacks remain. For instance, a drawback of term-to-concept matching is its inability to disambiguate terms, so annotators that inherit this method usually match terms with several possibilities. This drawback is encountered in the use of the NCBO annotator [9], and one way to solve this problem is to have several algorithms that use knowledge-based dictionaries to transform ambiguous terms into meanings that are clear for the annotator. These algorithms should also be able to correct incorrect grammar usage and wrong spellings by matching dictionary terms with similar spellings or phrases.

### Challenges of semantic annotation tools

Diverse tools are used in semantic annotation [1]. These tools also encounter some challenges, a few of which are listed below:

‒ Speed: This is one of the most common challenges. Annotations performed on huge datasets can take a lot of time to process.

‒ Language specificity: Most annotators are in English, which makes it difficult to apply semantic annotation in other languages.

‒ Document genre genericity: Annotators that support document input can face the problem of having to annotate different document formats, and not supporting a particular format could be a challenge.

‒ Text variation: According to Jovanovic and Bagheri [5], challenges are faced also due to the fact that there are different kinds of biomedical texts and variations in texts, for example between biomedical and clinical texts.

‒ Entity disambiguation: Entities mentioned in biomedical texts sometimes do not have enough context to disambiguate them.

These challenges and others are been studied, and many experts have tried to figure out ways to tackle them in newly developed systems. It may not be possible to fully resolve these challenges, but they can be reduced, and the following section shows how we tackled some of these challenges in the system developed for this project.

## Overview of the Table2Annotation Tool

In this section, we describe the proposed solution to build an ontology matching system. Our solution uses the NCBO annotator web service API for primary information retrieval.

The NCBO annotator annotates data with the MGrep term-to-concept matching tool and retrieves sets of annotations that are later expanded using various methods of semantic matching, meaning that this annotator goes through two stages. This annotator is unique because of the method it uses to associate concepts, instead of looking for the concept that best matches the provided context. This annotator uses BioPortal [10] and although it does not support disambiguation of terms, it is suitable for real-time processing. This annotator is available for free and is implemented through web services. This annotator is currently used in AgroPortal [11] and BioPortal.

The flow of Table2Annotation is quite simple and understandable. The system starts by taking an input dataset (CSV, Excel, etc.) and then processes the file by reading the data and fetching the necessary data to be annotated. It takes the necessary data and calls an external API provided by AgroPortal to annotate the data. The results returned from this process are processed by taking the Uniform Resource Identifier (URI), concept ID, and the matched words. Finally the annotated terms are saved and written to an output file for the user to access.

The operation of the matching system is described diagrammatically in Supplementary Fig. 1. In building this Table2Annotation tool we decided to use the NCBO annotator (AgroPortal API) to support the annotation of terms.

### Important algorithms

As discussed in the challenges section, there are several problems that must be dealt with. Thus, we developed specific algorithms to handle some of them.

#### Threading

First of all, the system was created in a functional independent approach where the major functions are independent. For example, obtaining inputs and annotation are independent. This allows us to better handle the slower part of the system. The function that slows down the system is the one that deals with iterating through the cells, taking the cell data, and then annotating this data. To reduce the problem of speed, we decided to create an algorithm to speed up the process. The algorithm uses the concept of multi-threading, allowing the function to be run by several processors (threads) concurrently.

#### Permutation

As discussed above regarding the problem of discontinuous entities, although this issue has not been fully resolved and future enhancements remain to be made, the problem of conjunctions can be reduced by creating an algorithm to handle this case.

#### Multiple dataset formats

The problem of document genre genericity was reduced by creating an algorithm to detect the format of the file being input by the user and then handling the process depending on the file format.

### Running Table2Annotation

Table2Annotation is a Java-based program that is currently executed through the command line interface. The user must have a dataset that he or she wants to annotate first. Table2Annotation is compiled after the code and all the functions explained in the previous section have been fully implemented. The compilation of Table2Annotation is done with all the necessary libraries included in the Java project. To run the system the user needs to input the following parameters: *input file* (mandatory), *column* (mandatory), *suggestions* (optional), *slice* (optional), *separator* (optional), and *sheet* (optional).

First, the user provides the path to the input file (dataset) and then provides the name of the column to be annotated. These two parameters are mandatory and the others are optional.

The other functions that can be passed as parameters are as follows: (1) suggestions (recommendations) of ontologies, allowing the user to specify which AgroPortal (or BioPortal) ontologies to use for the annotation process; (2) the slice (grouping), allowing the user to define which slice to use for the annotation process (slices can be compared to an instance of AgroPortal or BioPortal for a defined subset of ontologies); (3) the separator, if the file is a separated file type, allowing the user to define the type of separator used to split the cells; and (4) the sheet number if the file is an Excel file with multiple sheets.

After the command is executed, the system starts processing and stops when the process is completed. The results of this operation are output to a file in same format as the input file and given to the user.

## Results

In this section, we describe the results obtained from the Table2Annotation tool. We also describe the context of obtaining the results and an evaluation of the system.

**Fig. 1.**
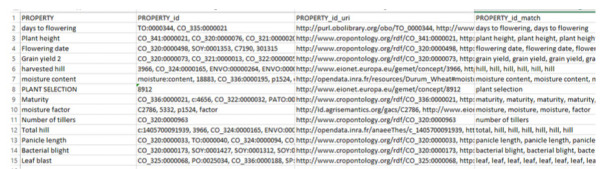
Dataset result without using suggestion and slice parameters. The column PROPERTY_ID shows all the matching ontologies.

First of all, we needed a dataset to test the tool, as shown in Fig. 1. The dataset that we used was quite small, as using a small dataset better demonstrates how the results are obtained, but the same principles are applied when using a large dataset. The dataset contains a “*PROPERTY*” column, which contains the terms to be annotated.

### Test without recommendations or slices

In this test, we ran the system without giving recommendations or slice options (i.e., an ontology list to map on provided by AgroPortal), and the results are shown in Fig. 1. In the results obtained by processing, we can see that there are three new columns: “*PROPERTY_id*”, “*PROPERTY_id_url,*” and “*PROPERTY_id_match.*” The first added column contains the concept IDs obtained from the annotation, the second added column contains the URIs of the concepts, and the third added column contains the matching of the terms with the concept.

### Test with a slice

**Fig. 2.**
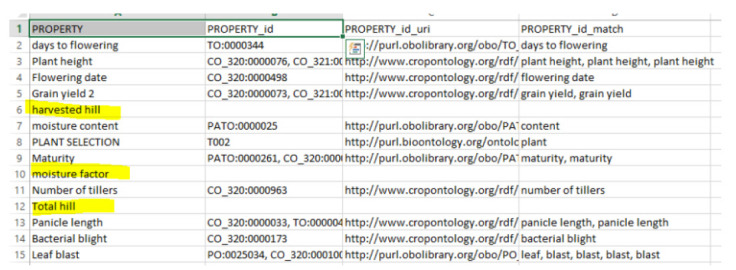
Dataset result using the slice parameter which filtered out some ontologies. The column named PROPERTY_ID shows less ontology matching than the one in Fig. 1.

In this test, we test-ran the system by giving it a slice called “*agrold*”, which contains ontology groups for agronomy. The results of the test are shown in Fig. 2. In the results, we can see three terms (highlighted in yellow) that do not match with any concept, because they do not have ontologies belonging to the “*agrold*” group.

### Test with recommendations

**Fig. 3.**
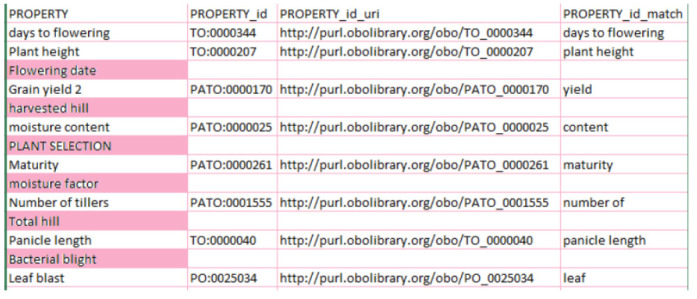
Dataset result using the recommendation parameter and keeping the three best ontologies annotating the dataset. The column named PROPERTY_ID shows less ontology matching than the one in Figs. 1 and 2.

In this test, we ran the system with three suggestion parameters: “*PO (Plant Ontology*)”, “*TO (Plant Trait Ontology*)”, and “*PATO (Phenotypic Quality Ontology)*.” The results of the test are shown in Fig. 3. In the results, we can see that six terms (highlighted in pink) had no matching concepts, because we filtered the annotation to the three ontologies given in the suggestions.

### Test with a permutation algorithm

**Fig. 4.**
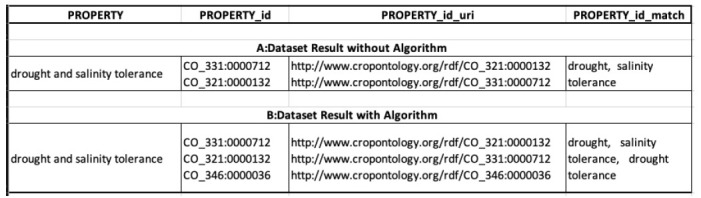
Comparison between splitting algorithm and without.

In this section, we tried to show the effect of having an algorithm to solve the problem of conjunctions in terms, which was mentioned earlier. We annotated the term “*drought and salinity tolerance*” and Fig. 4 shows the results. Fig. 4 (A, dataset result without algorithm) shows the results from the operation without the algorithm, and we can see that there are only matches for “*drought*” and “*salinity tolerance.*” Fig. 4 (B, dataset result with algorithm) shows the results from the operation with the algorithm, which yields matches for three terms: “*drought*”, “*salinity tolerance*”, and “*drought tolerance.*”

## Conclusion

In conclusion, Table2Annotation has strengths in certain criteria such as speed, error handling, and concept matching. First, we use a multi-threading algorithm that runs the process very effectively and efficiently. Second, it handles errors and exceptions by ignoring them whenever they occur. If there is an error while matching one term, it skips the term with an error and continues to the next one. If there is a general error, it still completes the matching process, but returns empty results. This method of error handling allows the user to run the process while multitasking and return to obtain the results without having to worry about system process terminations. Last, the matching results are good, and we see that cases of conjunctions are handled appropriately, so that the results contain more matches. The filters (slice and suggestions) also help to tailor the results to match the user’s expectations.

The system has strengths, but also has some weaknesses, such as relying on an internet connection and being dependent on the API. The system uses an external API, which can cause problems. Firstly, the system cannot work offline as it needs internet access to call the external API, which could be seen as a weakness. Secondly, if the external API is down for some reason, the system cannot be used. These weaknesses can be solved by building a full annotation system that does not depend on the availability of any external annotation API.

In the future, we think that we can improve algorithms to handle grammar problems and disambiguation. These algorithms should use language dictionaries to be able to transform terms without meaning (short forms) to something understandable to improve the process of matching to concepts. For example, when an abbreviated term is encountered, there should be a dictionary to look up the term and return the full meaning. This will further help to reduce the problems of spelling and grammar mentioned earlier.

## Supplementary Materials

Supplementary Table 1. Operation Diagram for Table2Annotation tool

## References

[1] Oliveira P, Rocha J Semantic annotation tools survey.

[2] Liao Y, Lezoche M, Panetto H, Boudjlida N Why, where and how to use semantic annotation for systems interoperability.

[3] Cooper L, Meier A, Laporte MA, Elser JL, Mungall C, Sinn BT (2018). The Planteome database: an integrated resource for reference ontologies, plant genomics and phenomics. Nucleic Acids Res.

[4] Wilkinson MD, Dumontier M, Aalbersberg IJ, Appleton G, Axton M, Baak A (2016). The FAIR Guiding Principles for scientific data management and stewardship. Sci Data.

[5] Jovanovic J, Bagheri E (2017). Semantic annotation in biomedicine: the current landscape. J Biomed Semantics.

[6] Bossy R, Golik W, Ratkovic Z, Bessieres P, Nedellec C BioNLP shared Task 2013: an overview of the bacteria biotope task.

[7] Bossy R, Deleger L, Chaix E, Ba M, Nedellec C Bacteria Biotope at BioNLP Open Shared Tasks 2019.

[8] Baumgartner W, Bada M, Pyysalo S, Ciosici MR, Hailu N, Pielke-Lombardo H CRAFT Shared Tasks 2019 overview: integrated structure, semantics, and coreference.

[9] Jonquet C, Shah NH, Musen MA (2009). The open biomedical annotator. Summit Transl Bioinform.

[10] Noy NF, Shah NH, Whetzel PL, Dai B, Dorf M, Griffith N (2009). BioPortal: ontologies and integrated data resources at the click of a mouse. Nucleic Acids Res.

[11] Jonquet C, Toulet A, Arnaud E, Aubin S, Dzale Yeumo E (2018). AgroPortal: a vocabulary and ontology repository for agronomy. Comput Electron Agric.

