# Supplementary figures and images for: Enabling a fast annotation process with the Table2Annotation tool
Source: Genomics Inform. 2020 Jun 15;18(2):e19. doi: 10.5808/GI.2020.18.2.e19 (PMC7362945; doi:10.5808/GI.2020.18.2.e19)

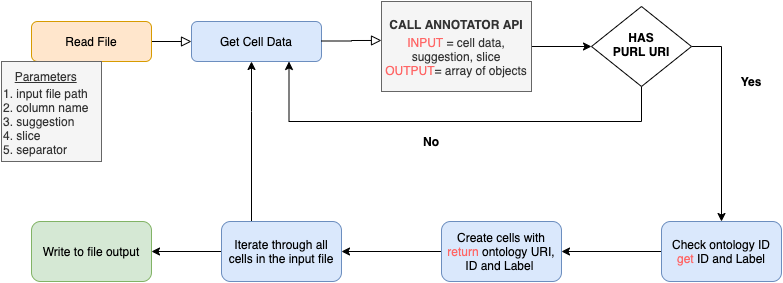


S1: Operation Diagram for Table2Annotation tool

Supplement: Supplementary Table 1. — Operation Diagram for Table2Annotation tool [file gi-2020-18-2-e19-suppl1.docx]
